# Primary Care Physician Use and Frequency of Visits Among Physicians in Ontario, Canada

**DOI:** 10.1001/jamanetworkopen.2022.27662

**Published:** 2022-08-19

**Authors:** Emily Rhodes, Claire Kendall, Robert Talarico, Elizabeth Muggah, Caroline Gerin-Lajoie, Christopher Simon, Taylor McFadden, Daniel Myran, Manish M. Sood, Peter Tanuseputro

**Affiliations:** 1 Ottawa Hospital Research Institute, Ottawa, Ontario, Canada; 2 Bruyère Research Institute, Ottawa, Ontario, Canada; 3 Department of Family Medicine, Institute du Savoir Montfort, Ottawa, Ontario, Canada; 4 Institute for Clinical Evaluative Sciences, Ottawa, Ontario, Canada; 5 Faculty of Medicine, University of Ottawa, Ottawa, Ontario, Canada; 6 Physician Wellness and Medical Culture Team, Canadian Medical Association, Ottawa, Ontario, Canada; 7 Department of Psychiatry, The Ottawa Hospital, Ottawa, Ontario, Canada; 8 Division of Nephrology, Department of Medicine, The Ottawa Hospital, Ottawa, Ontario, Canada; 9 Department of Family Medicine, The Ottawa Hospital, Ottawa, Ontario, Canada

## Abstract

**Importance:**

Maintaining a healthy physician workforce includes the routine use of primary care physician (PCP) services; however, physicians may face barriers to attaining formal care.

**Objective:**

To analyze access to and frequency of visits to PCPs among physicians compared with nonphysicians.

**Design, Setting, and Participants:**

This population-based, retrospective cohort study used registration data from the College of Physicians and Surgeons of Ontario, Canada, from January 1, 1990, to March 31, 2018. Data for all newly practicing physicians as of March 31, 2018, were linked to Ontario health administrative databases. Data were analyzed from August 25, 2020, to August 6, 2021.

**Main Outcomes and Measures:**

The main outcomes were enrollment in a PCP practice and visits with a PCP. Generalized estimating equations compared primary care visits between physicians and nonphysicians, matched 1:5 based on age, sex, neighborhood income quintile, and health region.

**Results:**

Among 19 581 physicians (mean [SD] age, 43.99 [8.94] years; 53.27% male) matched to 97 905 nonphysicians, physicians were less likely to be enrolled with a PCP than were nonphysicians (81.8% vs 86.4%; absolute difference, 4.6%; adjusted odds ratio [OR], 0.75; 95% CI, 0.72-0.79) and had fewer primary care visits during the preceding 2 years (median [IQR], 2 [0-4] vs 4 [1-7]; adjusted relative rate ratio [RRR], 0.59; 95% CI, 0.58-0.60). Physicians aged 40 years or older and male physicians were less likely to be rostered (ages 40-44 years: OR, 0.70 [95% CI, 0.64-0.77]; male: OR, 0.60 [95% CI, 0.57-0.63]) and more likely to have a lower frequency of PCP visits (ages 40-44 years: RRR, 0.53 [95% CI, 0.51-0.56]; male: RRR, 0.50 [95% CI, 0.50-0.51]) compared with nonphysicians.

**Conclusions and Relevance:**

In this retrospective cohort study, enrollment with a PCP practice and frequency of visits were lower among physicians compared with a matched general population of nonphysicians. Individual, system, and medical cultural factors associated with these results need to be better understood so that physicians can take better care of themselves and their patients.

## Key Points

**Question:**

Do physicians access primary care physicians as patients, and if so, how frequently?

**Findings:**

In this cohort study of 19 581 physicians and 97 905 matched nonphysicians in Ontario, Canada, physicians were less likely to be enrolled with a primary care physician and used primary care services less frequently than nonphysicians.

**Meaning:**

In this study, physicians were less likely to access primary care services compared with nonphysicians, suggesting that improved understanding of individual, system, and medical cultural factors associated with these findings is needed to improve health for physicians and their patients.

## Teaser

This cohort study investigates access to and frequency of visits to primary care physicians among physicians compared with the general population in Ontario, Canada.

## Introduction

Primary care is an essential part of the health care system. Primary care services are intended to be the first point of contact for new medical concerns; to provide long-term, comprehensive patient-centered care; and to coordinate specialist care.^1,2^ When primary care is routinely accessed, it has been shown to contribute to prevention of disease, reduced hospital readmissions, and appropriate care for complex chronic illnesses, and it is considered cost-effective.^3^ When seeking care for themselves, physicians have unique circumstances in that they may be more aware of their medical condition and may have greater access to primary care physicians (PCPs) and specialists. It is unknown, however, how this knowledge translates to health-seeking behavior for both primary and specialist-based care among physicians.

Some physicians face barriers to accessing care, including time constraints, confidentiality concerns, and stigma.^4^ A perceived pressure to remain healthy to maintain the trust of their patients and respect of their colleagues may lead physicians to seek informal care or “hallway medicine” (ie, obtaining prescriptions or informal advice from colleagues) from their peers.^5,6^ Survey and qualitative data have shown that the number of physicians registered with a PCP ranges from 20% to 100%, with most studies reporting higher proportions in regions such as the UK, where it is mandatory to register with a PCP.^4^ To our knowledge, there have not been any studies examining physicians’ access to PCPs using objective health administrative data. We sought to close that gap by linking registry data of 19 581 physicians in Ontario, Canada, to health administrative data, thereby addressing access to PCPs and frequency of visits among physicians compared with a matched general population of nonphysicians at a population level.

## Methods

Regional ethics board approval was obtained from the Ottawa Hospital Research Institute. Because only deidentified data were analyzed, individual-level informed consent was waived. The study followed the Strengthening the Reporting of Observational Studies in Epidemiology (STROBE) reporting guideline.

### Design and Setting

This study was part of a broader initiative, Health Evaluation and Liveliness for Physicians Through Meaningful Data (HELP-MD), that aimed to access and analyze large-scale, secondary epidemiological data to study the health and wellness of physicians. We conducted a retrospective cohort study of all Ontarian physicians compared with a matched cohort of the general population. Physicians were identified by newly registering with the College of Physicians and Surgeons of Ontario (CPSO), a licensing requirement for practicing medicine, between January 1, 1990, and March 31, 2018. We used unique, encoded identifiers to conduct deterministic and then probabilistic data linkage based on name, date of birth, and sex to link physicians from the database of the CPSO to health administrative databases housed at ICES. Additional details on our probabilistic matching were previously published.^7^

### Delivery of Primary Care in Ontario

In Ontario, patients are enrolled or “rostered” to a PCP who is responsible for providing comprehensive primary care services to those patients; the patient, in turn, only seeks care from their specified PCP unless they have an emergency.^8^ This system of PCP rostering is widely used internationally; improves patient follow-up, continuity of care, and accountability; and is associated with improved clinical outcomes.^9,10^ In Canada, formal primary care for adults is provided by family physicians, and a referral is required to see an internal medicine specialist.

### Data Sources

Physician demographic and specialty information were obtained from CPSO registration data. Relevant data were obtained from encoded, linked databases housed at ICES (eTable 1 in the Supplement). Additional physician specialty information was obtained from the ICES physician database, which uses billing and workforce information to assign specialties based on the proportion of specialty billing codes used. Primary care was defined by the Primary Care Population Database, which is an ICES-derived, population-level data set that includes all people in Ontario who are deemed eligible at the index date. An eligible person was an Ontario resident who was alive on March 31, 2018; had at least 1 contact with the health care system within 9 years of the index date; and had Ontario Health Insurance Plan eligibility. Each quarterly data set includes a basic demographic variable—information on primary care registration identifying a patient’s attachment status (rostered, virtually rostered, or not rostered or not in the patient enrollment model) along with other variables, such as emergency department visits, hospitalization, access to specialty care, continuity of care, and models of care.

### Study Population

All physicians newly registered with the CPSO and nonphysicians aged 25 years or older on March 31, 2018, were included in the study cohort (eTable 2 in the Supplement). The age cutoff was chosen because it is the average age of completion of medical school in Canada. Physicians were considered inactive if they had billed less than 10% of the days in the year before March 31, 2018, and were therefore not eligible for the study. Although primary care use differs by age, sex, and place of residence, physicians were matched 1:5 to the general population based on age, sex, neighborhood income quintile, and local health integration network (1 of 14 health administrative zones in Ontario).

### Covariates

We obtained physician information, including age, sex, geographic health region, location of residence (rural or urban), income quintile, primary specialty (family medicine, psychiatry, anesthesia, internal medicine, surgery, radiology, missing, or other), and comorbidity (hypertension, heart failure, acute coronary syndrome, chronic obstructive pulmonary disease, asthma, diabetes, and mental health diagnosis). We defined a mental health diagnosis as having 1 or more visits for a mental health diagnostic code in the previous 2 years or a visit to a psychiatrist for any cause. For other chronic conditions, we used validated *International Statistical Classification of Diseases and Related Health Problems, Tenth Revision* codes and/or physician billing algorithms, with a minimum of a 5-year historical period.^11,12^

### Outcomes

Our outcomes were enrollment with a PCP, defined as being rostered, and frequency of visits. Rostering was defined using the most responsible physician assigned by the Client Agency Program Enrollment (CAPE) as of March 31, 2018 (eTable 3 in the Supplement). The most responsible physician is defined as the regulated health care physician who has overall responsibility for directing and coordinating the care of a patient.^13^ The CAPE data set identified patients enrolled in different primary care models in Ontario. For patients not in CAPE, we used an alternative method to identify primary care patterns using physician billing in which patients are allocated to a PCP on the basis of the largest dollar amount of core primary care services for that patient in the previous 2 years.^14^ This is termed “virtual rostering,” a method with 85% agreement with CAPE after validation.^14^ For study outcome ascertainment, we examined the 2-year period before the index date for evidence of rostering (either CAPE or virtual) and the frequency of PCP visits.

### Statistical Analysis

We examined and compared baseline characteristics of the physicians and matched nonphysicians from the general population using means or medians for continuous variables and proportions or frequencies for categorical variables. To examine the association of physician characteristics with likelihood of PCP rostering, we used logistic regression using generalized estimating equations with an exchangeable covariance structure to account for the matched study design. In addition to matching, models were adjusted for place of residence (rural vs urban) and comorbidity (hypertension, heart failure, acute coronary syndrome, chronic obstructive pulmonary disease, asthma, diabetes, and mental health diagnosis) as covariates. We further examined models for subgroups of interest using interaction terms. These models included residence and comorbidity covariates as well as physician type (PCP, surgeon, internal medicine physician, anesthesiologist, radiologist, psychiatrist, and other) and primary care use compared with matched individuals from the general population. We repeated the same models using negative binomial regression to examine the frequency of PCP visits. Odds ratios (ORs) and relative rate ratios (RRRs) with 95% CIs were reported for physicians compared with the general population. We conducted all analyses with SAS software, version 7.1 (SAS Institute Inc). We deemed 2-sided *P* < .05 as statistically significant. All analyses were conducted at ICES.

## Results

### Demographic and Clinical Characteristics

Our study cohort consisted of 19 581 physicians practicing in Ontario, matched to 97 905 nonphysicians in the general population. Among physicians, the mean (SD) age at baseline was 43.99 (8.94) years, 53.2% were male, and 53.5% lived in the highest income quintile neighborhoods. Overall, all comorbidities were less frequent among physicians compared with matched nonphysicians. Mental health diagnoses were the most frequent condition among physicians (16%), followed by asthma (9.4%) and hypertension (8.3%), and all mental health diagnoses were less frequent among physicians than in the matched general population (Table 1 and eTable 4 in the Supplement). Across physician specialties, PCPs were youngest (mean (SD) age, 43.39 [9.52] years) and had the highest proportion of female physicians (4856 of 9059 [53.6%]); surgery and other specialties had the lowest proportions of female physicians (902 of 2697 [33.4%] and 113 of 340 [33.2%], respectively). More radiologists resided in the highest income neighborhoods (467 of 681 [68.6%]), and PCPs had the lowest proportion of physicians living in the highest income neighborhoods (4368 of 9059 [48.2%]). Mental health visits in the past 2 years were most frequent among psychiatrists (333 of 1170 [28.5%]), followed by PCPs (1564 of 9059 [17.3%]) and internal medicine physicians (677 of 4672 [14.5%]), and the lowest frequency of mental health visits in the past 2 years was observed among surgeons (288 of 2697 [10.7%]) (eTable 5 in the Supplement).

**Table 1.** Baseline Characteristics of Physicians and a Matched Sample From the General Population^a^

Characteristic	Physicians (n = 19 581)^b^	General population (n = 97 905)^b^
Age, y		
Mean (SD)	43.99 (8.94)	43.99 (8.94)
25-29	382 (2.0)	1910 (2.0)
30-34	2780 (14.2)	13 900 (14.2)
35-39	3724 (19.0)	18 620 (19.0)
40-44	3697 (18.9)	18 485 (18.9)
45-49	3711 (19.0)	18 555 (19.0)
≥50	5287 (27.0)	26 435 (27.0)
Sex		
Female	9171 (46.8)	45 855 (46.8)
Male	10 410 (53.2)	52 050 (53.2)
Income quintile^c^		
1	1199 (6.1)	5995 (6.1)
2	1864 (9.5)	9320 (9.5)
3	2429 (12.4)	12 145 (12.4)
4	3618 (18.5)	18 090 (18.5)
5	10 471 (53.5)	52 355 (53.5)
Rural^d^	906 (4.6)	8970 (9.2)
Mental health visit in the past 2 y	3124 (16.0)	21 685 (22.1)
Comorbidities		
Hypertension	1634 (8.3)	13 033 (13.3)
Heart failure	45 (0.2)	371 (0.4)
Myocardial infarction	48 (0.2)	545 (0.6)
COPD	122 (0.6)	3275 (3.3)
Asthma	1840 (9.4)	12 547 (12.8)
Diabetes	681 (3.5)	6204 (6.3)

Abbreviation: COPD, chronic obstructive pulmonary disease.

^a^ Physicians and nonphysicians from the general population were matched 1:5 based on age, sex, income quintile, and local health integration network.

^b^ Data are reported as number (percentage) of individuals unless otherwise indicated.

^c^ An income quintile of 1 is the lowest, and 5 is the highest.

^d^ Location of physician residence vs urban.

### PCP Rostering and Frequency of Visits Compared With the General Population

Overall, 81.8% of physicians were rostered to a PCP compared with 86.4% matched members of the general population (absolute difference, 4.6%) (Table 2). Compared with the general population, physicians were more likely to be virtually rostered (18.2% vs 13.6%) and less likely to be CAPE rostered (68.6% vs 77.5%). In adjusted models, physicians had 25% lower odds of being rostered (OR, 0.75; 95% CI, 0.72-0.79) compared with the general population. Furthermore, the median number of PCP visits by physicians per year was lower compared with the general population (2 [IQR, 0-4] vs 4 [IQR, 1-7]), and this was consistent after covariate adjustment (RRR, 0.59; 95% CI, 0.58-0.60) (Table 2).

**Table 2.** Outpatient Primary Care Rostering and Visit Frequency Among Physicians Compared With Matched Nonphysicians From the General Population^a^

	Physicians (n = 19 581)	General population (n = 97 905)	Crude point estimate (95% CI)^b^	Adjusted point estimate (95% CI)^b^
Primary care rostering, No. (%)	16 022 (81.8)	84 595 (86.4)	0.70 (0.68-0.74)	0.75 (0.72-0.79)^c^
Frequency of primary care visits, median (IQR)	2 (0-4)	4 (1-7)	0.53 (0.52-0.55)	0.59 (0.58-0.60)^d^

^a^ Rostering and frequency were ascertained by evidence of primary care visits among study participants in the 2-year period before the index date. Physicians and nonphysicians from the general population were matched 1:5 based on age, sex, income quintile, and local health integration network.

^b^ Odds ratio shown for rostering data; relative rate ratio shown for frequency of visits data.

^c^ Generalized estimating equation with 1:5 physician-to-nonphysician matching adjusted for practice location (rural and urban) and comorbidities (hypertension, myocardial infarction, congestive heart failure, chronic obstructive pulmonary disease, asthma, diabetes, and mental health diagnosis).

^d^ Negative binomial regression with 1:5 physician-to-nonphysician matching adjusted for practice location (rural and urban) and comorbidities (hypertension, myocardial infarction, congestive heart failure, chronic obstructive pulmonary disease, asthma, diabetes, and mental health diagnosis).

When examining physician characteristics associated with PCP use (Table 3) and frequency of visits (Table 4), physicians aged 40 years or older were less likely to be rostered (40-44 years: OR, 0.70 [95% CI, 0.64-0.77]; 45-49 years: OR, 0.63 [95% CI, 0.57-0.69]; ≥50 years: OR, 0.55 [95% CI, 0.51-0.60]; *P* < .001 for interaction) and more likely to have a lower frequency of visits (40-44 years: RRR, 0.53 [95% CI, 0.51-0.56]; 45-49 years: RRR, 0.52 [95% CI, 0.50-0.55]; ≥50 years: RRR, 0.53 [95% CI, 0.51-0.55]); *P* < .001 for interaction) compared with the general population. Male physicians were significantly less likely to be rostered (OR, 0.60; 95% CI, 0.57-0.63) and had a lower frequency of visits (RRR, 0.50; 95% CI, 0.50-0.51) compared with the general population. Higher income quintiles were also associated with lower rostering among physicians compared with the general population. Absence of a mental health diagnosis or asthma was associated with less rostering among physicians compared with the general population. Psychiatrists were more likely to be rostered (88.9%; OR, 1.25; 95% CI, 1.04-1.50) compared with the general population, and all remaining specialty physicians were similarly or less likely to be rostered compared with the general population.

**Table 3.** Characteristics Associated With PCP Rostering Among Physicians Compared With Matched Nonphysicians From the General Population^a^

Characteristic	Physicians, No. (%) (n = 16 022)	General population, No. (%) (n = 84 595)	Adjusted odds ratio (95% CI)^b^	*P* value
Age, y				
25-29	338 (2.11)	1574 (1.86)	1.72 (1.23-2.39)	<.001
30-34	2380 (14.85)	11 511 (13.61)	1.28 (1.14-1.44)	
35-39	3141 (19.60)	15 910 (18.81)	0.95 (0.86-1.05)	
40-44	3001 (18.73)	16 011 (18.93)	0.70 (0.64-0.77)	
45-49	2983 (18.62)	16 236 (19.19)	0.63 (0.57-0.69)	
≥50	4179 (26.08)	23 353 (27.61)	0.55 (0.51-0.60)	
Sex				
Female	8243 (51.45)	40 853 (48.29)	1.17 (1.08-1.26)	<.001
Male	7779 (48.55)	43 742 (51.71)	0.60 (0.57-0.63)	
Income quintile^c^				
1	937 (5.85)	4770 (5.64)	1.01 (0.87-1.18)	<.001
2	1502 (9.37)	7755 (9.17)	0.92 (0.81-1.05)	
3	2022 (12.62)	10 324 (12.20)	0.93 (0.82-1.05)	
4	2930 (18.29)	15 836 (18.72)	0.64 (0.58-0.71)	
5	8631 (53.87)	45 910 (54.27)	0.70 (0.66-0.74)	
Place of residence^d^				
Rural	732 (4.57)	7568 (8.95)	0.89 (0.74-1.06)	.053
Urban	15 282 (95.38)	77 015 (91.04)	0.75 (0.72-0.78)	
Mental health diagnosis				
Yes	2877 (17.96)	64 636 (23.59)	1.05 (0.92-1.21)	<.001
No	13 145 (82.04)	19 959 (76.41)	0.73 (0.70-0.76)	
Comorbidities				
Hypertension				
Yes	1425 (8.89)	11 967 (14.15)	0.66 (0.56-0.78)	.12
No	14 597 (91.11)	72 628 (85.85)	0.76 (0.73-0.80)	
Heart failure				
Yes	37 (0.23)	345 (0.41)	0.47 (0.20-1.14)	.88
No	15 985 (99.77)	84 250 (99.59)	0.76 (0.73-0.79)	
Myocardial infarction				
Yes	41 (0.26)	502 (0.59)	0.64 (0.28-1.49)	.72
No	15 981 (99.74)	84 093 (99.41)	0.76 (0.72-0.79)	
COPD				
Yes	105 (0.66)	2998 (3.54)	0.55 (0.32-0.94)	.29
No	15 917 (99.34)	81 597 (96.46)	0.76 (0.73-0.79)	
Asthma				
Yes	1636 (10.21)	11 229 (13.27)	1.09 (0.93-1.28)	<.001
No	14 386 (89.79)	73 366 (86.73)	0.73 (0.70-0.77)	
Diabetes				
Yes	581 (3.63)	5631 (6.66)	0.64 (0.50-0.80)	.16
No	15 441 (96.37)	78 964 (93.34)	0.76 (0.73-0.79)	
Physician specialty				
Family	7546 (47.10)	NA	0.81 (0.76-0.86)	NA
Internal medicine	3790 (23.65)	NA	0.73 (0.68-0.79)	NA
Surgery	2023 (12.63)	NA	0.55 (0.50-0.60)	NA
Psychiatry	1040 (6.49)	NA	1.25 (1.04-1.50)	NA
Anesthesia	808 (5.04)	NA	0.94 (0.79-1.12)	NA
Radiology	540 (3.37)	NA	0.68 (0.56-0.82)	NA
Other	275 (1.72)	NA	0.77 (0.59-1.01)	NA

Abbreviations: COPD chronic obstructive pulmonary disease; NA, not applicable; PCP, primary care physician.

^a^ Rostering was ascertained by evidence of primary care visits among study participants in the 2-year period before the index date. Physicians and nonphysicians from the general population were matched 1:5 based on age, sex, income quintile, and local health integration network.

^b^ Generalized estimating equation with 1:5 physician-to-nonphysician matching adjusted for practice location (rural and urban) and comorbidities (hypertension, myocardial infarction, congestive heart failure, COPD, asthma, diabetes, and mental health diagnosis).

^c^ An income quintile of 1 is the lowest, and 5 is the highest.

^d^ Data missing for less than 0.1%.

**Table 4.** Characteristics Associated With Frequency of PCP Visits by Physicians Compared With Matched Nonphysicians From the General Population^a^

Characteristic	Physicians, median (IQR)	General population, median (IQR)	Adjusted relative rate ratio (95% CI)^b^	*P* value
Age, y				
25-29	3 (1-5)	3 (1-7)	0.76 (0.68-0.85)	<.001
30-34	2 (1-5)	3 (1-7)	0.76 (0.73-0.80)	
35-39	2 (0-4)	3 (1-7)	0.64 (0.61-0.67)	
40-44	1 (0-3)	3 (1-7)	0.53 (0.51-0.56)	
45-49	1 (0-3)	3 (1-7)	0.52 (0.50-0.55)	
≥50	2 (0-4)	4 (2-8)	0.53 (0.51-0.55)	
Sex				
Female	3 (1-5)	5 (2-9)	0.67 (0.66-0.69)	<.001
Male	1 (0-2)	3 (1-6)	0.50 (0.50-0.51)	
Income quintile^c^				
1	2 (0-4)	4 (1-8)	0.57 (0.53-0.62)	.01
2	2 (0-4)	4 (1-8)	0.60 (0.56-0.64)	
3	2 (0-4)	4 (1-7)	0.62 (0.58-0.65)	
4	2 (0-4)	4 (1-8)	0.61 (0.59-0.64)	
5	2 (0-3)	4 (1-7)	0.57 (0.55-0.58)	
Place of residence^d^				
Rural	1 (0-3)	2 (1-5)	0.62 (0.56-0.68)	.24
Urban	2 (0-4)	4 (1-7)	0.58 (0.57-0.60)	
Mental health diagnosis				
Yes	4 (2-7)	7 (4-12)	0.58 (0.56-0.60)	.33
No	1 (0-3)	3 (1-6)	0.59 (0.58-0.60)	
Comorbidities				
Hypertension				
Yes	3 (1-5)	6 (3-11)	0.58 (0.55-0.62)	.83
No	2 (0-4)	3 (1-7)	0.59 (0.57-0.60)	
Heart failure				
Yes	3 (1-7)	7 (4-13)	0.69 (0.46-1.03)	.47
No	2 (0-4)	4 (1-7)	0.59 (0.57-0.60)	
Myocardial infarction				
Yes	3 (1-5)	5 (3-11)	0.68 (0.47-0.98)	.48
No	2 (0-4)	4 (1-7)	0.59 (0.57-0.60)	
COPD				
Yes	2 (1-5)	6 (3-10)	0.56 (0.44-0.71)	.69
No	2 (0-4)	4 (1-7)	0.59 (0.57-0.60)	
Asthma				
Yes	2 (1-5)	5 (2-9)	0.62 (0.58-0.66)	.07
No	2 (0-4)	3 (1-7)	0.58 (0.57-0.59)	
Diabetes				
Yes	3 (1-7)	7 (4-12)	0.62 (0.57-0.68)	.17
No	2 (0-4)	3 (1-7)	0.58 (0.57-0.60)	
Physician specialty				
Family	2 (1-4)	NA	0.70 (0.68-0.71)	NA
Internal medicine	1 (0-3)	NA	0.51 (0.49-0.53)	NA
Surgery	1 (0-2)	NA	0.39 (0.37-0.42)	NA
Psychiatry	2 (1-5)	NA	0.65 (0.62-0.70)	NA
Anesthesia	1 (0-3)	NA	0.48 (0.44-0.51)	NA
Radiology	1 (0-3)	NA	0.46 (0.42-0.50)	NA
Other	1 (0-3)	NA	0.48 (0.42-0.54)	NA

Abbreviations: COPD, chronic obstructive pulmonary disease; NA, not applicable; PCP, primary care physician.

^a^ Frequency was ascertained by evidence of primary care visits among study participants in the 2-year period before the index date. Physicians and nonphysicians from the general population were matched 1:5 based on age, sex, income quintile, and local health integration network.

^b^ Negative binomial regression with 1:5 physician-to-nonphysician matching adjusted for practice location (rural and urban) and comorbidities (hypertension, myocardial infarction, congestive heart failure, COPD, asthma, diabetes, and mental health diagnosis).

^c^ Lowest income quintile is 1; highest is 5.

^d^ Data missing for less than 0.1%.

## Discussion

A healthy physician workforce requires timely and ongoing high-quality primary care. However, to our knowledge, few studies have objectively examined PCP use among physicians. In this population-based cohort study of 19 581 physicians practicing in Ontario, we found that physicians were less likely to be registered with a PCP and to have fewer visits in the preceding 2 years compared with a matched cohort of 97 905 individuals from the general population. Furthermore, PCP registration and frequency of visits differed based on key physician characteristics compared with the general population. Primary care physician registration after adjustment was lower among physicians aged 40 years or older and among males but higher among psychiatrists and physicians with a mental health diagnosis or asthma.

Previous studies about receipt of primary care by physicians demonstrated a high degree of regional variability, with estimates ranging from 21% among Swiss PCPs to 96% to 100% in the UK, where registration with general practitioners is mandatory.^4,15^ A 2017 Canadian Medical Association survey of physicians and residents found that 82% reported having a PCP, consistent with the current study.^16,17^ Unlike our population-based study using health administrative databases, most previous studies used self-reported surveys with variable response rates, were limited to selected cohorts (eg, graduates of an individual program or hospital system), and had various selection biases (eg, language limitations, concerns about confidentiality, or differential response rates by sex, age, and physician type).^18,19^ These factors substantially limited the ability to assess PCP use fully and appropriately among physicians, especially at the population level. Furthermore, our results provide insight into the frequency of PCP visits by physicians—a preexisting knowledge gap.

Our quantitative assessment of primary care use and frequency of receipt provides insight. Despite recommendations by governing physician groups, physicians were significantly less likely to be rostered with a PCP and attended fewer primary care visits than comparable members of the general population.^16,18^ Although physicians in general often benefit from better physical health compared with the general population, this characteristic may limit the preventive aspect of primary care and may result in missed or inappropriate screening and early detection opportunities.^20,21^ This is consistent with a recent Ontario study of colorectal cancer screening among physicians in which physicians were more likely to bypass stool occult blood testing, the recommended first-line screening test, and proceed directly to a more invasive colonoscopy.^22^ Conversely, with their medical knowledge, physicians may be less likely to seek medical care inappropriately, such as for a self-resolving illness, and thus, the optimal amount and frequency of physician-to-PCP interactions remain unclear.

Primary care physician use and uptake differed by key physician characteristics. Older physicians were less likely to interact with PCPs compared with matched nonphysicians from the general population. This finding may be related to healthier aging, ease, and access to informal consultation and/or generational differences in accepting and seeking medical help. Male physicians were less likely to interact with primary care in general. A previous study^23^ suggested that male physicians were more likely to self-treat acute or chronic conditions and to order self-bloodwork for diagnosis or monitoring compared with female physicians. In the general population, women are more likely to see a PCP for obstetrical and gynecological care compared with men, a factor that also likely contributed to our findings. Psychiatrists were the most likely to be rostered, showing large discrepancies among the physician types. Previous studies identified hours worked, work intensity and flexibility, attitudes toward health care or primary care and the perception of illness, acceptance and culture of self-care, and concerns of confidentiality as key barriers among physicians.^19,24^ Of interest, physicians who practiced in rural areas, who may face isolation and compounded confidentially issues, did not differ in primary care rostering or visit frequency compared with physicians living in nonrural areas. This finding suggests that opportunities to engage in primary care were not associated with physical travel distances or stigma.

Our findings may directly inform health care organizations, such as wellness programs, involved with physicians about how to improve primary care use. Quantifying results showing that physicians are less likely to use primary care than the general population is a complex task owing to the use of hallway medicine among physicians. Use of hallway medicine may have contributed to our results and may be an area of deficiency in that these health care encounters are fast and incompletely captured. A previous study^4^ estimated this form of self-care to occur among one-third to nearly all physicians studied. Lack of primary care may limit access to health care, worsen documentation, and most importantly, reduce the delivery of evidence-based health care.^2^

Improvements and solutions for primary care use among physicians will require a multilevel approach. At the individual level, the practice of self-care and optimization of physical and mental health should be perceived as essential and an investment toward improved performance or provision of patient care. This includes enhanced self-awareness and insight to better identify stressors and be able to practice skills and access resources to mitigate their impact and maintain wellness. At the system level across health care organizations and faculties of medicine, there should be raised awareness of the importance of accessing primary care, the availability of easy and rapid access to PCPs, and protected time and coverage available so that physicians can access their appointments. From the first day of medical school, the culture should encourage a model of healthy physicians that acknowledges the high pressures of the environment, promotes the concept of PCP involvement to enhance resiliency and performance, and decreases the stigma around accessing health care. The shift in medical culture could also help to reduce the guilt that physicians may feel toward their patients or colleagues when they need to cancel patient care and ask for clinical coverage. There also should be a review of the perception of a healthy physician compared with a physician who optimally manages their health. In addition, an emerging body of evidence supports an association between physicians’ individual health practices and the delivery of improved care to their patients.^20,22,25^

### Limitations

The current study has several limitations. We aimed to quantify registered and captured health care encounters billed within the Ontario health system and, as such, could not quantitate self-care practices or hallway medicine. Many physicians may partake in such activities; however, the extent and quality of care remain unclear.^5^ We focused on formal enrollment with PCPs, which may not reflect the full scope of care services (eg, psychologists or chiropractors) or the use of fragmented, single-use health care (eg, walk-in clinics). Comorbidities and mental health diagnoses are more likely to be identified among those rostered with a PCP and may represent a source of bias. Physicians were more likely to be virtually rostered, defined based on billing practices, with a potential for misclassification. We examined a 2-year period for evidence of PCP rostering and frequency of visits and did not account for individuals who changed or discontinued a PCP practice. More granular data on lifestyle, habits, physician practice characteristics, and setting were not available. Because this was an observational study, we were unable to assess causality, and the reported observations require confirmation in future investigations.

## Conclusions

In a large cohort of physicians from Ontario, we found lower PCP rostering and frequency of visits among physicians compared with the general population, with key differences noted by sex, age, and physician type. Emphasis on the importance and improvement of access to primary care for physicians is a potential means to improve overall health for physicians and patients.

## Notes

Supplement. **eTable 1.** Administrative Health Databases Housed at ICES **eTable 2.** Study Cohort Creation **eTable 3.** Virtual Rostering Algorithm **eTable 4.** Local Health Integration Networks (LHINs) by Physicians and a Matched (5 to 1) Sample From the General Population **eTable 5.** Baseline Characteristics for Total Physicians, by Physician Specialty, and a Matched (5 to 1) Sample From the General Population
