# Supplementary material for: Primary Care Physician Use and Frequency of Visits Among Physicians in Ontario, Canada
Source: JAMA Netw Open. 2022 Aug 19;5(8):e2227662. doi: 10.1001/jamanetworkopen.2022.27662 (PMC9391953; doi:10.1001/jamanetworkopen.2022.27662)
Supplement: Supplement. — eTable 1. Administrative Health Databases Housed at ICES eTable 2. Study Cohort Creation eTable 3. Virtual Rostering Algorithm eTable 4. Local Health Integration Networks (LHINs) by Physicians and a Matched (5 to 1) Sample From the General Population eTable 5. Baseline Characteristics for Total Physicians, by Physician Specialty, and a Matched (5 to 1) Sample From the General Population [file jamanetwopen-e2227662-s001.pdf]

Supplemental Online Content

Rhodes E, Kendall C, Talarico R, et al. Primary care physician use and frequency of visits among physicians in Ontario, Canada. *JAMA Netw Open*. 2022;5(8):e2227662.
doi:10.1001/jamanetworkopen.2022.27662

eTable 1. Administrative Health Databases Housed at ICES
eTable 2. Study Cohort Creation
eTable 3. Virtual Rostering Algorithm
eTable 4. Local Health Integration Networks (LHINs) by Physicians and a Matched (5 to 1) Sample From the General Population
eTable 5. Baseline Characteristics for Total Physicians, by Physician Specialty, and a Matched (5 to 1) Sample From the General Population

This supplemental material has been provided by the authors to give readers additional information about their work.

© 2022 Rhodes E et al. *JAMA Network Open*.

eTable 1. Administrative Health Databases Housed at ICES

Database	Description	Variables
College of Physicians and Surgeons of Ontario (CPSO)	Regulatory college for the medical profession. Physicians must register and renew their membership yearly to practice in Ontario.	• Specialty<br>• Medical school<br>• Level of training<br>• License restrictions
Discharge Abstract Database (DAD)	Administrative, clinical, and demographic data on all hospital discharges in Ontario.	• Deliberate self-harm<br>• Mental health conditions
ICES Physicians Database (IPDB)	Comprises information from the Ontario Health Insurance Plan (OHIP), Corporate Provider Database (CPDB), the Ontario Physician Human Resource Data Centre (OPHRDC) database, and the OHIP database of physician billings. The CPDB contains information on physician demographics, specialty training and practice location. The data in the CPDB is validated against the OPHRDC database, which verifies physician specialty and practice location information through periodic telephone interviews with physicians.	• Specialty
Ontario Health Insurance Plan (OHIP) Claims Database	Physician billings: Claims for physicians in Ontario – includes claims in both inpatient and outpatient settings.<br><br>Non-physician billings: Health professionals for provincial insured services, such as select midwives, oral surgeons, chiropractors, optometrists, and physiotherapists. Some care may occur for inpatients.	• Mental health conditions

© 2022 Rhodes E et al. *JAMA Network Open*.

	Laboratory: Outpatient laboratory services. Does not include laboratory services for inpatients.	
ORGD vital statistics	Obtains information about mortality from death certificates which are completed by physicians. All deaths within Ontario are registered in the office of the division registrar within which the death occurs.	• Completed suicide
Registered Persons Database (RPDB)	Data on individuals registered under OHIP and who are eligible for the Ontario Drug Program.	• Age<br>• Sex

© 2022 Rhodes E et al. *JAMA Network Open*.

eTable 2. Study Cohort Creation

Number of Physician Records in CPSO from 1990 to 2018	55,537
Number of Physician Records with valid unique identified from 1990 to 2018	46,623
Number of Physicians (keeping first registration date) from 1990 from 2018	45,835
Number of Physicians alive and eligible March 31 2018	24,952
Number of Physicians who billed on 10% of days between April 1 20017 to March 31 2018	19,629
Number of Physicians with non-missing LHIN, income quintile and >=25 on March 31 2018	19,581
Final Cohort Physician Age, sex, income quintile and LHIN matched to non-phys. 1:5 on March 31 2018	97, 905[a]

© 2022 Rhodes E et al. *JAMA Network Open*.

eTable 3. Virtual Rostering Algorithm

**Variable**	**Definition**
Virtual rostering	For patients that were not in CAPE a virtual rostering methodology was applied<br><br>a. All visits to specialist were obtained= 00, 05, 26 for the 2-year period preceding the index date for the following fee codes- A001, A003, A007, A903, E075, G212, G271, G372, G373, G365, G538, G539, G590, G591, K005, K013, K017, P004, A261, K267, K269 – core Primary Care codes<br><br>b. Cost of services (cost × number of service) was derived by linking to standard pricing file<br><br>c. For each patient the highest billing physician was selected

© 2022 Rhodes E et al. *JAMA Network Open*.

eTable 4. Local Health Integration Networks (LHINs) by Physicians and a Matched (5 to 1) Sample From the General Population

Baseline Characteristics	Physicians<br>N= 19,581	General Population*<br>N= 97,905
LHIN1	577 (2.9%)	2,885 (2.9%)
LHIN2	1,507 (7.7%)	7,535 (7.7%)
LHIN3	802 (4.1%)	4,010 (4.1%)
LHIN4	1,964 (10.0%)	9,820 (10.0%)
LHIN5	396 (2.0%)	1,980 (2.0%)
LHIN6	1,850 (9.4%)	9,250 (9.4%)
LHIN7	4,366 (22.3%)	21,830 (22.3%)
LHIN8	2,606 (13.3%)	13,030 (13.3%)
LHIN9	877 (4.5%)	4,385 (4.5%)
LHIN10	798 (4.1%)	3,990 (4.1%)
LHIN11	2,335 (11.9%)	11,675 (11.9%)
LHIN12	519 (2.7%)	2,595 (2.7%)
LHIN13	663 (3.4%)	3,315 (3.4%)
LHIN14	321 (1.6%)	1,605 (1.6%)

* Matched 1:5 based on age, sex, income quintile and LHIN; % percentage, SD standard deviation

© 2022 Rhodes E et al. *JAMA Network Open*.

eTable 5. Baseline Characteristics for Total Physicians, by Physician Specialty, and a Matched (5 to 1) Sample From the General Population

Baseline Characteristics	Total Physicians N= 19,581	Family N=9,059	Medicine N=4,672	Surgery N=2,697	Psychiatry N=1,170	Anaesthesia N=962	Radiology N=681	Other N=340	General population N=97, 905[a]
Age at index Mean (SD)	43.99 (8.94)	43.39 (9.52)	43.64 (8.18)	44.37 (8.18)	46.78 (9.72)	45.19 (7.89)	44.90 (7.54)	47.09 (8.18)	43.99 (8.94)
Sex N (%)									
Female	9,171 (46.8)	4,856 (53.6)	2,161 (46.3)	902 (33.4)	573 (49.0)	331 (34.4)	235 (34.5)	113 (33.2)	45,855 (46.8)
Male	10,410 (53.2)	4,203 (46.4)	2,511 (53.7)	1,795 (66.6)	597 (51.0)	631 (65.6)	446 (65.5)	227 (66.8)	52,050 (53.2)
Income Quintile N (%)									
1	1,199 (6.1)	598 (6.6)	281 (6.0)	150 (5.6)	81 (6.9)	37 (3.8)	29 (4.3)	23 (6.8)	45,855 (46.8)
2	1,864 (9.5)	937 (10.3)	427 (9.1)	232 (8.6)	113 (9.7)	80 (8.3%)	40 (5.9)	35 (10.3)	52,050 (53.2)
3	2,429 (12.4)	1,254 (13.8)	536 (11.5)	284 (10.5)	155 (13.2)	115 (12.0)	54 (7.9)	31 (9.1)	12,145 (12.4)
4	3,618 (18.5)	1,902 (21.0)	797 (17.1)	431 (16.0)	214 (18.3)	141 (14.7)	91 (13.4)	42 (12.4)	5,995 (6.1)
5	10,471 (53.5)	4,368 (48.2)	2,631 (56.3)	1,600 (59.3)	607 (51.9)	589 (61.2)	467 (68.6)	209 (61.5)	9,320 (9.5)
Rural	906 (4.6)	700 (7.7)	64 (1.4)	97 (3.6)	17 (1.5)	13 (1.4)	14 (2.1)	<6	8,970 (9.2)
Mental Health Visits N (%)	3,124 (16.0)	1,564 (17.3)	677 (14.5)	288 (10.7)	333 (28.5)	130 (13.5)	89 (13.1)	43 (12.6)	21,685 (22.1)
Comorbidities N (%)									
Hypertension	1,634 (8.3)	718 (7.9)	349 (7.5)	228 (8.5)	151 (12.9)	94 (9.8)	51 (7.5)	43 (12.6)	13,033 (13.3)
Heart failure	45 (0.2)	23 (0.3)	7 (0.1)	7 (0.3)	6	<6	<6	<6	371 (0.4)
MI	48 (0.2)	18 (0.2)	7 (0.1)	11 (0.4)	7 (0.6%)	<6	<6	<6	545 (0.6)
COPD	122 (0.6)	53 (0.6)	29 (0.6)	16 (0.6)	13 (1.1)	<6	7 (1.0)	<6	3,275 (3.3)
Asthma	1,840 (9.4)	936 (10.3)	440 (9.4)	222 (8.2)	108 (9.2)	65 (6.8)	55 (8.1)	14 (4.1)	12,547 (12.8)
Diabetes	681 (3.5)	328 (3.6)	147 (3.1)	80 (3.0)	77 (6.6)	22 (2.3)	11 (1.6)	16 (4.7)	6,204 (6.3)

© 2022 Rhodes E et al. *JAMA Network Open*.

[a]Matched 1:5 based on age, sex, income quintile and local health integration network (LHIN); % percentage, SD standard deviation, MI myocardial infarction, COPD chronic obstructive pulmonary disorder
Cell sizes <6 were suppressed as per ICES policy to prevent reidentification

© 2022 Rhodes E et al. *JAMA Network Open*.
